# An Architecture for the Performance Management of Smart Healthcare Applications

**DOI:** 10.3390/s20195566

**Published:** 2020-09-28

**Authors:** Andressa Vergütz, Nelson G. Prates, Bruno Henrique Schwengber, Aldri Santos, Michele Nogueira

**Affiliations:** Department of Informatics, Federal University of Paraná, Curitiba 80060-000, Brazil; avergutz@inf.ufpr.br (A.V.); ngpjunior@inf.ufpr.br (N.G.P.J.); bhschwengber@inf.ufpr.br (B.H.S.); aldri@inf.ufpr.br (A.S.)

**Keywords:** smart healthcare, reliability, latency, network slicing, network-as-a-service, 6G

## Abstract

The sixth-generation (6G) network intends to revolutionize the healthcare sector. It will offer smart healthcare (s-health) treatments and allow efficient patient remote monitoring, exposing the high potential of 6G communication technology in telesurgery, epidemic, and pandemic. Healthcare relies on 6G communication technology, diminishing barriers as time and space. S-health applications require strict network requirements, for instance, 99.999% of service reliability and 1 ms of end-to-end latency. However, it is a challenging task to manage network resources and applications towards such performance requirements. Hence, significant attention focuses on performance management as a way of searching for efficient approaches to adjust and tune network resources to application needs, assisting in achieving the required performance levels. In the literature, performance management employs techniques such as resource allocation, resource reservation, traffic shaping, and traffic scheduling. However, they are dedicated to specific problems such as resource allocation for a particular device, ignoring the heterogeneity of network devices, and communication technology. Thus, this article presents PRIMUS, a performance management architecture that aims to meet the requirements of low-latency and high-reliability in an adaptive way for s-health applications. As network slicing is central to realizing the potential of 5G–6G networks, PRIMUS manages traffic through network slicing technologies. Unlike existing proposals, it supports device and service heterogeneity based on the autonomous knowledge of s-health applications. Emulation results in Mininet-WiFi show the feasibility of meeting the s-health application requirements in virtualized environments.

## 1. Introduction

Computational intelligence and the Internet of Things (IoT) evolve along with the advances of next-generation wireless technologies, such as 5G and 6G cellular networks [1]. This evolution has boosted the development of traditional healthcare into the Internet of Medical Things (IoMT) and smart healthcare (s-health) services [2]. S-health is essential to both patients and healthcare practitioners. Once it improves treatments, it enables the remote patient monitoring, enhances the patient quality of life, and reduces costs. Forecasts show a growing market for s-health of 12.5% until 2025 [3]. However, s-health applications include a wide range of services, such as critical care monitoring and remote surgery, requiring low-latency and high-reliability communication to transmit data to healthcare professionals promptly and patients [4,5]. Managing network resources may improve reliability and latency, and it seems to be an efficient way to achieve s-health requirements. If low-latency and high-reliability are not achieved, s-health services are degraded, leading even to their denial.

High-reliability means successful data transmission without extrapolating the maximum latency supported by the s-health application [6]. Achieving the performance requirements is not a trivial task since s-health applications rely on a heterogeneous communication environment. Regulatory institutions (e.g., the U.S. Food and Drug Administration) reinforce the development of reliable health devices and applications [7] by acts as the Health Insurance Portability and Accountability Act (HIPAA). Furthermore, the network traffic diversity has grown exponentially due to 5G, the advent of 6G, and IoMT smart scenarios (e.g., smart hospital) that daily generate a massive amount of data, resulting in high complexity to support s-health application requirements. The variety of s-health applications (e.g., telemedicine, critical care monitoring) makes the task even more complicated since each application has specific requirements [8]. For instance, remote surgery requires reliability of 99.999% and latency of 1 ms, while continuous monitoring of noncritical patients support 125 ms of latency [5,9]. Performance management leads to improvements in s-health applications by adjusting or tuning network resources (e.g., bandwidth, and memory) to application needs, once the improper use of network resources results in latency and reliability degradation, and unsatisfactory Quality of Service.

The high level of reliability demanded by s-health applications reveals limitations in traditional network mechanisms (e.g., priority queues) given traffic diversity and volume [8,10,11]. Current network technologies are not prepared to deal with such amount of data and devices, since they do not support the dynamics and on-demand network resource allocation. For instance, the IEEE 802.11 standard has four access categories to give priority to different types of traffic (e.g., voice, video) [12]. Nonetheless, health-related traffic uses the same category as voice traffic. Another work has created a special priority for medical alerts [13,14,15,16]. However, the latency required by s-health application is not yet achieved. The recent network slicing paradigm is a potential solution for performance management on s-health since it contains essential features, such as automation, fast adaptation, and customization [5]. Although such concept is addressed in the literature [15,17,18,19], the implementation of a realistic network slicing environment is often not presented. Moreover, solutions that tailor network resources (e.g., define a fixed amount of resources) to transmit only health data packets are unique to one type of application [13,14]. Therefore, it is indispensable new ways to manage the amount of network traffic, adapt network resources (e.g., change the link used by the application according to the bandwidth available), reach specific requirements, and then support s-health [20].

As the emerging concept of network slicing is central to realizing 5G–6G networks [18], we advocate for network slicing as a potential solution to manage and achieve the s-health application and service requirements (e.g., high-reliability and low-latency). Network slicing employs virtualization techniques (Network Function Virtualization—NFV, Software Defined Networks—SDN) for heterogeneous environments, encompassing cellular network and IoMT, once it allows the same physical network to be split into isolated virtual subnetworks [5,6]. The benefits of network slicing for s-health include autonomous traffic analysis and network resources adaptation to specific requirements [10]. Therefore, we propose an architecture for the PeRformance management of smart healthcare applIcations requireMents based on network resoUrce Slicing (PRIMUS) that manages traffic and adapts network resources to support s-health. PRIMUS creates network slices for the applications. It adapts the network resources by creating and selecting dynamic routes for s-health traffic. The creation of these routes has metrics the flow latency and packet loss rate, being based on the application traffic identification, proposed in the previous work [20,21].

The PRIMUS architecture was evaluated on Mininet-WiFi Network Emulator [22] that supports virtualization techniques since network slicing requires such technologies. The simulation comprises an emulated wireless network infrastructure containing two switches, one access point, three wired devices, three wireless devices, and three servers. The network was virtualized and sliced based on Openflow rules by Libera Hypervisor [23], and managed by ONOS. We have considered three slice types involving s-health synthetic traffic and Best Effort (BE). Each device generated traffic simulating each slice type and behavior to a server by the iPerf tool. Results show the feasibility of achieving high-reliability and low-latency in the s-health context by performance management. In the network slices created for s-health, the latency has reached values around 10 ms.

This article proceeds as follows. Section 2 overviews the related works. Section 3 details the background on virtualization technologies and network slicing. Section 4 presents the proposed performance management architecture, network model, and assumptions. Section 5 presents the performance evaluation and discusses results. Finally, Section 6 concludes the article.

## 2. Performance Management Approaches

Facing the issues and difficulties regarding to achieve high-reliability and low-latency, few approaches have been proposed in the last years [24]. We have identified well-known mechanisms to attain application/service requirements in the literature, such as resource reservation, traffic shaping, traffic separation, scheduling (a queuing mechanism), resource allocation, and the most recent network slicing. Each mechanism presents advantages and limitations. Sometimes, these mechanisms are combined to achieve specific requirements. As this article aims to achieve s-health requirements, we discuss these mechanisms under the perspective of their feasibility in the s-health context. Hence, in this section, we review the main approaches, proposals and explain their characteristics and drawbacks. A summary of the works is given in Table 1. We classify the works based on the considered approaches, the towards requirements, and implementation environments in the table.

A couple of approaches have been introduced for a few decades, and they usually include resource reservation and traffic shaping. Resource reservation is the most basic and static approach, and it requires communication between the applications and the network, indicating the requirements. For instance, the well-known Resource ReSerVation Protocol (RSVP), employed by Integrated Services (IntServ) [25], signalizes the requirements for each device, allowing single receivers to change channels and optimize bandwidth. However, such approach is not scalable since it needs to install multiple states about the reservations in each router. Internet providers commonly use traffic shaping since it controls the amount of data and matches the network traffic to the client-hired profile. For instance, it coordinates resource consumption by applications, such as torrents. When a given application affects the performance of others, its traffic is delayed. However, such approach is specific for clients or one application type, not being scalable and flexible.

Nowadays, traffic separation and scheduling mechanisms predominate [12,13,14,15,26,27,28]. Traffic separation classifies the network traffic according to the application or device that generated it, such as Differentiated Services (Diffserv) [39]. Scheduling follows a queue mechanism (e.g., first in, first out) to transmit the packets according to some priority rule. Network traffic monitoring employs traffic separation since it analyzes the traffic to learn about application/device behavior. For instance, there are studies using machine learning techniques (ML-techniques) to manage network traffic in the 5G network and achieve requirements [26,27]. Hence, the authors have employed ML-techniques to learn about network traffic to adjust some resources and enable various policy-driven network management. However, they have proposed only an abstraction-level architecture [26]. Although the authors have applied virtualization techniques to enable network management, they have considered only mobile applications from smartphones, not scalable for different scenarios [27].

Moreover, in general, the studies rely on network technology. For instance, for IoT, Li et al. [28] proposed a service-oriented scheduling model based on application and network layers classified into delay-sensitive and best-effort categories. Wi-Fi, by the Enhanced Distributed Channel Access (EDCA), modifies some parameters (e.g., control window) to give priority in the medium access. EDCA has four access categories, including voice and video [12]. However, nowadays, there is a more significant variety and diversity of types of applications, ranging from streaming or game to healthcare. Hence, such works do not support a wide range of applications. Vergutz et al. [13] created a new access category, especially to medical alerts. The results achieved around 200 ms of latency for medical alerts. S-health applications with restricted requirements do not support such latency. Therefore, improvements are crucial to achieve specific requirements.

In a cellular network context, there are studies concerned about critical/sensitive applications (e.g., alerts and mission critical). Lloret et al. [14] presented a 4G–5G architecture for s-health that classifies data vital into normal and abnormal by ML-techniques. Abnormal data receives a high priority. Petrov et al. [15] presented a 5G virtualization-based architecture for end-to-end reliability of mission-critical traffic in an urban scenario. Through virtualization techniques, the architecture gives priority for all mission-traffic over other types of traffic. Results showed a cost since the high data rate of critical sessions brings degradation to other users’ services. Wang et al. [16] proposed a scheduler algorithm and filtered more relevant healthcare data by different levels of priority. Although the authors have reduced the loss rate, the latency is still high. Moreover, these studies did not consider the management and customization of abnormal or critical data from different users. The architectures are not adaptable and easy-customized for different scenarios.

In traffic separation, conventional technologies, such as virtual local area networks (VLAN), provide isolated networks for a specific application. However, in VLAN, it is difficult to control the availability and performance guarantee since the number of users operating in the network affects the entire system performance [6]. Therefore, the approaches mentioned above are still insufficient to accommodate the diversity of smart application requirements. Based on the literature, there is a diversity of network performance techniques due to unique network properties. Some applications cannot communicate their requirements, and the mapping between application requirements and network capabilities is not standard [38]. Hence, the appearance of some mechanism configurable between the applications and the network presents itself as a compelling alternative. Moreover, network virtualization can help achieve requirements since it controls virtual resources and supports the diversity of application, service, network control, and management [6]. The new paradigm, network slicing, is based on network virtualization. It has a considerable potential to achieve requirements over heterogeneous networks, especially for s-health applications that employ different network technologies in their communication [40]. Next, we present the most relevant works using network slicing in performance management context.

### Network Slicing Approaches

The emergent network slicing technology has been used in numerous studies for network performance management. Unlike static and exclusive policies, network slicing creates slices (sub-networks) by virtualization techniques over the same physical infrastructure and allows users to share virtual network resources. There are main types of slices, such as ultra-reliable and low-latency communications (URLLC) and enhanced mobile broadband (eMMB). S-health applications, such as remote surgery, require URLLC between wearables and infrastructure networks [1,4]. However, network slicing impact on reliability and latency performance has been limited, and improving reliability methods has not been investigated on s-health [10]. Generally, studies on network slicing implement some network slice type, such as URLLC, following some priority queuing [17,29,30,31], or present an end-to-end architecture [19,37].

There are works combining network slicing with another performance management mechanism. For instance, Ge [17] proposed a network slicing solution with priority queuing to provide ultra-reliability and low-latency in vehicular networks. The authors created three URLLC slices to control vehicle-state reports (e.g., speed, and location), event-drive (e.g., emergency), and user information. Kalor et al. [29] used network slicing with priority queuing to manage the different requirements, such as reliability, low latency, and high data rates. Considering a personalized medicine manufacturing use case, the authors created three levels: static telegram allocation (traffic from one application), shared telegram (traffic from several applications), and telegram overwriting (applications with higher priority overwrite network traffic). Both works improved reliability and latency. However, user density increased the latency, especially in vehicle context. Moreover, the studies only presented a mathematical model, not implementing any experiment with virtualization techniques.

Yilmaz et al. [30] presented the challenges for mission-critical machine type communication and proposed to use orthogonal frequency-division multiplexing. Network simulations were presented, achieving 3 ms of latency for one link direction (not latency end-to-end). Although the author mentioned URLLC, they did not use network slicing and any virtualization technique. Virtual network resources are crucial to share, adapt, and allocate resources flexibly. Karimi et al. [31] presented a scheduling mechanism for URLLC and eMBB by exploiting the radio channel time-frequency variations. The authors have considered an urban 5G scenario. For load with 4–8 Mbps and 14 Mbps, the method reached 1.5 ms and 4 ms of latency, respectively. However, they have sliced the physical layer increasing considerable complexity. Virtualization techniques can be more adaptable and low-cost. Kurtz et al. [18] and Bouzidi [32] presented a network slicing approach for critical communications on 5G and end-to-end delay. Based on network function virtualization, the authors proposed a queuing driven approach focusing on different applications. However, it was only considered a smart grid as a critical communication, abstaining from healthcare applications. Moreover, although virtualization techniques improve flexibility and automation, some works pointed out that, to efficiently manage network resources and meet performance requirements, network function virtualization techniques need mechanisms to predict performance degradation [41]. Packet classification also assists network functions to determine which action they should take for each incoming packet [42]. Therefore, improvements in virtualization techniques also are necessary to manage network resources efficiently.

Sciancalepore et al. [33,34] proposed an admission control algorithm, and a scheduler to assign priority queue. The authors analyzed the network traffic per slice and defined six traffic classes, where streaming receives the highest priority level. In a second moment [34], the authors proposed a slice as a service to assist the slice optimization and orchestration. They decided on schedule or rescheduled slices based on the traffic analyzed. For instance, when there are mission-critical messages, other slices are rescheduled to support the critical requirements. However, the authors did not consider a real scenario since the streaming traffic did not has more priority traffic than other traffic (e.g., healthcare). Moreover, it is not clear the type of traffic rescheduled to support priority traffic. Richart et al. [36] presented an allocation resource mechanism. The mechanism aims to slice the WiFi Access Point by considering the transmission time (airtime) as the resource. The authors identified the existing traffic flows, assigned a queue for each flow, and scheduled the traffic following the round-robin scheduling. Through network simulations, the mechanism allocated the airtime resource successfully. However, it was not presented performance metrics results (e.g., latency). Moreover, they did not consider the slice types (e.g., URLLC) and did not implement the traffic identification, only the scheduler.

Salhab et al. [35] proposed a framework based on machine learning to identify the application requirements changes over time and achieve dynamic optimization. The authors considered multiple slices using different resources percentage from the total available resource pool. The results identified the traffic classes successfully, and the framework improved the throughput for eMBB traffic. However, the results are superficial since the authors only presented eMBB traffic results, abstaining from others, and the ML-techniques results only contain the accuracy, without network traffic details. In the context of network slicing with traffic shaping, Caballero et al. [43] tailored the network slices for each application, allowing flexible allocation of resources. Each application communicates its demands to the infrastructure and receives network resources. However, it is a crucial resource control mechanism since some applications can monopolize resources. Salvat et al. [37] and Marquez et al. [38] adopted a data-driven approach to quantify the efficiency of resource sharing. The authors adopted an overbooking policy, i.e., the operator allocates more resources than are actually available for the users, believing that some users will not use all resources allocated (they made an analogy with airplane and hotels companies). However, when some traffic does not receive resources, the packet data are considered best-effort traffic or is removed, increasing the packet losses. This approach is not suitable for the s-health scenario since critical applications do not support packet losses. Moreover, if applications do not follow agreements, some applications can request more resources than necessary, harming other applications. Husain et al. [19] presented a mobile edge architecture for IoT to customize network resources. They proposed positioning network slicing technology in the gateway, closer to the users, to minimize latency. However, the authors presented only abstraction-level architecture.

Based on the literature, there are several open issues related to network slicing since it is a novel concept. Several studies addressed the URLLC slice type, which involves critical applications with critical requirements, e.g., remote surgery and autonomous vehicles [17,29,31]. However, the studies addressed an individual characteristic of the network slicing concept, i.e., some studies addressed queuing priority. Other studies proposed a resource allocation method [44]. Nevertheless, network slicing can bring enormous benefits for s-health because it encompasses essential characteristics such as customization and adaptation. In this article, we propose an architecture for performance management in the s-health context based on network slicing.

## 3. Background

Standards organizations have defined network slicing in numerous ways [5,6,45]. In a nutshell, it refers to all the attempts to enable the availability of the network-as-a-service approach according to user demands khan:2020. Its key enabling technologies involve Software Defined Network (SDN) and Network Function Virtualization (NFV) due to programmability and flexibility in networking resource management [10,46]. Together, they enable dynamic resource monitoring, allocation, and isolation in the network infrastructure by a logically centralized software-based platform [47]. Thus, network slicing allocates bandwidth, spectrum, and computing capacity based on application requirements [40]. A tenant refers to a group of users accessing the shared resources with specific access privileges and access rights. It directly serves an end-to-end communication, or a vertical network tenant to access a service, under a fully qualified set of requirements [48].

**Figure 1:**
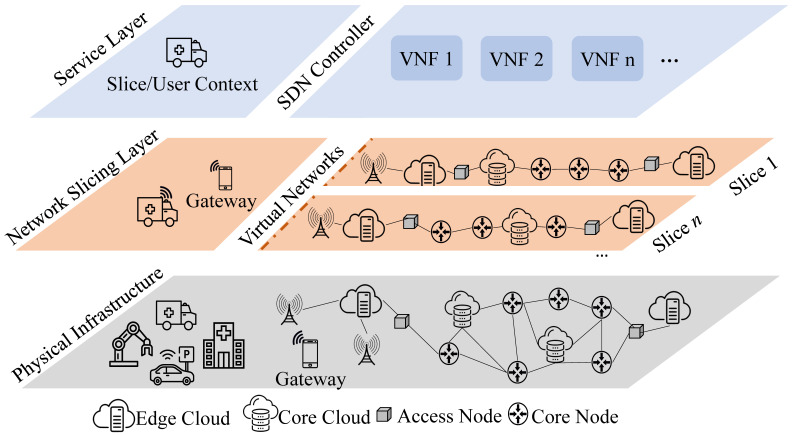
An overview of network slicing.

SDN technologies simplify the networking hardware by decoupling the control from the data plane. It removes control functionality from network devices (e.g., switches). These devices become simple packets forwarders (the data plane), and the network control becomes directly programmable via an open interface (e.g., OpenFlow). This configuration enables communication compatibility and interoperability among different data and control plane devices. The SDN devices forward the packets based on a network flow abstraction. Thus, the flow tables store a programmable set of forwarding rules in the memory architecture. SDN controllers, logically centralized, customize the flow rules on the memory and accommodate network control functions [47]. Therefore, SDN enables a high-level of slice abstraction from different network forwarding rules, dynamically managing network slices, and a simple NFV development. Figure 1 presents an overview of network slicing using NFV and SDN.

NFV defines a set of softwarized functions to enable the original network functions (NF) to share the same physical environment, i.e., dispatch a network function, such as a firewall, to a provider (cloud data center) as a software instance, i.e., Virtual Network Functions (VNFs). The detachment of software from hardware helps to reassign and to share the infrastructure resources, allowing the deployment of new network services faster over the same physical platform. Therefore, VNFs allow networks to be agile and capable of responding automatically to the needs of the services running over it. This comprises a crucial feature from network slicing once it involves multiple heterogeneous physical resources managed centrally.

## 4. The PRIMUS Architecture

This section introduces PRIMUS, *an architecture for ada**P**table sma**R**t healthcare appl**I**cations require**M**ents based on network Reso**U**rce **S**licing*. Unlike the literature, PRIMUS adapts network virtual resources and routes according to the smart healthcare application demands. Hence, it identifies the applications and configures the network on-the-fly. PRIMUS receives as input the application identified in a previous work [21], assisting our framework proposed in [20]. The next subsections detail the network model, the smart healthcare application requirements, and the PRIMUS architecture.

### 4.1. Network Model

PRIMUS considers applications with critical requirements, such as s-health intensive care and remote surgery. Both applications require low-latency and high-reliability. Reliability in this work means successfully transmitting and delivering packets without exceeding the maximum latency supported by the application [4,6]. However, unlike the literature, PRIMUS does not refrain from other applications such as telemedicine [49]. Table 2 presents the main s-health applications and requirements. These applications have been defined by the Next Generation Mobile Network Alliance (NGMN) [6] and 3rd Generation Partnership Project (3GPP) [5,45]. In general, almost all s-health applications require reliable communications since patient health comprises a critical use case [6]. The most flexible applications, such as fitness and calorie counting, have no stringent requirements. Body temperature and diabetes monitoring consist of daily continuous monitoring applications. These applications collect specific vital signs and issue medical alerts when critical situations occur (e.g., extreme body temperature values). They support up to 125 ms latency [9]. In contrast, telemedicine applications demand high data rates and low-latency (10 ms). Therefore, PRIMUS needs to meet these requirements automatically through network slicing.

**Figure 2:**
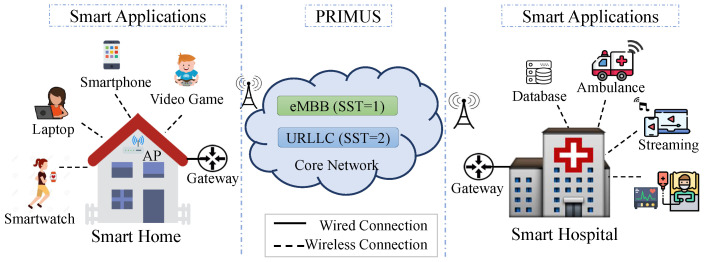
The PRIMUS network overview.

PRIMUS takes into account a network model, as illustrated in Figure 2. The model encompasses the communication between smart devices and environments, such as smart home and smart hospitals, supporting new applications, particularly s-health. In the smart environments, the network infrastructure contains different devices generating network traffic to a gateway by wireless connection (e.g., Wi-Fi, Bluetooth, 5G). The edge devices involve smartphones, augmented reality on video games, laptops, smartwatches from the smart home, ambulance, wearable, and sensors from the smart hospital. We can have usual devices in both smart scenarios, e.g., smartphones. Each device contains applications running on it. PRIMUS receives as input the application identification from our previous work [21]. In the core network, devices involve base stations, routers, switches, and others. Such network model supports network slicing and virtualization techniques (NFV and SDN).

Formally, let N=(1,…,n) be the set of tenants (e.g., end-users) that generates network traffic from a given application app, where App=(1,…,app) is a set of applications. Hence, let $v_{n,s}\left(t\right)$ be the traffic of an application associated with a slice *s* at tenant *n* during instant *t*. Each network slice is responsible for traffic messages exchanged between hosts, considering application requirements. Hence, the slice *s* needs to achieve a set of requirements $Q=\{q_{1},q_{2},\ldots ,q_{n}\}$ specific from the application app. A set of available network resources R=(1,…,r), such as bandwidth and computing, are necessary to achieve such requirements. Therefore, a given slice *s* is composed by virtual network resources $r_{1},r_{2}\ldots r_{n}$, network traffic $v_{n,s}\left(t\right)$ and an associated user *n* and application app that demands some requirement *q*. Moreover, each slice has one controller *c*, from the set of controllers C=(1,…,c), managing the resources. One slice can have more than one application and user associated at the same time. However, organizations have defined that at most eight network slices may serve one end-user at a time [5,6]. Thus, let $u_{num}$ be the respective user identification *u* using num network slices in parallel. When num≥8, PRIMUS removes the user *u* from the slice *s* and reallocates the resources for another slice. In case an app uses more resources than allowed, or it is harming other app, PRIMUS defines a threshold duration time $t_{d}$. Hence, the app has a timing $t_{d}$ until it makes the resource available. Otherwise, when exceeding $t_{d}$, PRIMUS removes the slice and the application associated, i.e., it removes the associated traffic, application, and user, making resources available.

PRIMUS marks all slice *s* created with the Slice/Service Type SST and Slice Differentiator SD. The SST refers to the network slice behavior in terms of features and services. There are three main types of SST: (1) Enhanced Mobile Broadband (eMBB) with high data rates, (2) Ultra-Reliable Low-Latency Communications (URLLC) with more critical data traffic, and (3) High-Density Massive Internet of Things (mIoT) with a massive amount of users and devices. Thus, s-health intensive care and remote surgery applications fit into the URLLC slice type. Based on this, the SST values are SST=1 for eMBB, SST=2 for URLLC, and SST=3 for mIoT. Note that URLLC slices can not be removed because it involves critical applications. Thus, when occurring resource overloading, PRIMUS reallocates network resources or, in extreme situations, removes a mIoT or eMBB slice. The SD is optional information that complements the SST to differentiate among multiple network slices of the same SST. For instance, SD serves to identify extreme critical care applications from typical s-health applications. Finally, PRIMUS has full visibility and control of the end-to-end slice and its performance to maintain the overall requirements of the applications. PRIMUS computes a set of performance variables P=(1,…,p) (e.g., packet loss rate and latency) to analyze the slice performance and create dynamic virtual routes. Such performance variables define the state of the physical structure for establishing or adapting network slices to meet predefined requirements.

### 4.2. PRIMUS Architecture

**Figure 3:**
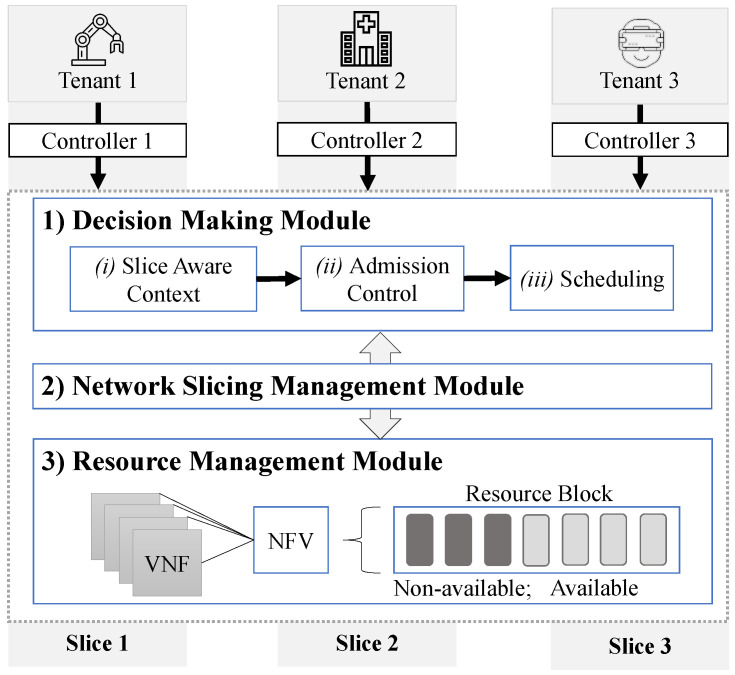
PRIMUS architecture.

Figure 3 illustrates the PRIMUS architecture that follows the modules: (1) Decision-Making, (2) Network Slice Management, and (3) Resource Management. PRIMUS considers the application context to create and allocate network slices. Thus, different tenants *u* (users) generate network traffic. Each one belongs to a use case and demands specific requirements *Q*. Tenant 1 ($u_{1}$), 2 ($u_{2}$) and 3 ($u_{3}$) refer to devices from industry 4.0, smart hospitals, and augmented reality, respectively. While tenant $u_{1}$ requires ultra-low-latency, tenant $u_{3}$ demands high bandwidth. Hence, PRIMUS needs to monitor the network traffic to establish and control the slice to support the requirements, especially the requirements of s-health. The identification and monitoring of s-health applications follow our previous work [20,21]. Based on the knowledge about the application context, PRIMUS creates the slices routes and adapts resources through NFVs. It enables devices to logically and separately share numerous network services without overloading switches. Therefore, PRIMUS considers the highest virtualization level, coordinating devices, network functions, and resources with greater flexibility. The next subsections detail the three modules of PRIMUS.

#### 4.2.1. Decision-Making Module

This module takes decisions on slice configuration, slice admission, and slice scheduling following three steps: (i) Slice-Aware Context, (ii) Admission Control, and (iii) Scheduling. These steps define the creation of a new slice or the allocation of a pre-configured slice. There are pre-configured slices for s-health since urgent care application supports only 1 ms of latency. Thus, the slice-aware context step gets information about the application context and its requirements. As aforementioned, our previous work [21] identifies the application that is generating network traffic. Hence, through announce control messages, PRIMUS informs the requirements demanded by each application (we detail control messages in Table 3 from Resource Management Module). Thus, the first step analyzes and stores this information to make future decisions. The information about the requirements enhances the network resources utilization efficiency, once the infrastructure provider can get the network slice demands on the slice-aware context. The slice-aware context stores data regarding applications, requirements, and end-users. It stores information about identical applications but from different end-users since several users may generate traffic, simultaneously. In such situations, PRIMUS allocates equal or the same network slice to meet the same application needs. Each slice allows up to eight users per time. The resources are further pre-allocated following the slice-aware to provide the admission control policy.

The admission control step needs information about the available resource and priority policy. Thus, it offers information to the scheduling step to analyze the priority levels and, jointly to resource management, to know the available and allocated resources. Moreover, for the admission control policy, we assume that the resource demands are accurate. Thus, in the first moment, the admission control analyzes the network slice type, i.e., URLLC, eMBB, or mIoT, to decide what network resource each slice will receive since URLLC has priority over eMBB and mIoT. The scheduling step stores and updates the priority information. As URLLC transmits critical network data, it receives the highest priority. eMBB and mIoT receive the second and third priorities, respectively. Hence, there are three priority queues: 1-URLLC, 2-eMBB, and 3-mIoT. Afterwards, to know the allocated and available resources, the admission control verifies resource management’s resource block. For URLLC, there are pre-configured slices to not increase the latency since slice creation and configuration take time.

#### 4.2.2. Network Slice Management Module

**Figure 4:**
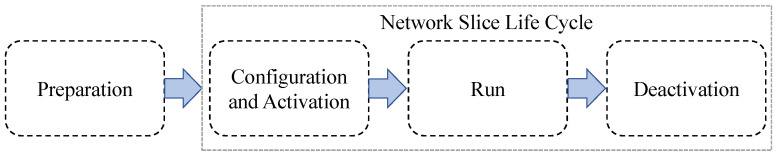
Network slice life cycle.

It comprises a fundamental component of network slicing, being responsible for managing and orchestrating network slices. It also allows, configures, and determines slice policies, such as restricted access by a given user, and configures the network resources used by it. Hence, the module has visibility and control of end-to-end slices and the performance of each slice. Thus, it controls the overall functioning of the slices. Moreover, it controls the slice life cycle, i.e., it controls the preparation, configuration, supervision, and deactivation phases, as illustrated in Figure 4.

The preparation phase consists of creating and verifying the network slice, since it prepares the necessary network environment to encompass the network slice created, such as virtual network functions. In addition, it verifies if there are running slices of the same type (e.g., eMBB and URLLC). Furthermore, it marks each slice with the Slice Differentiator SD to add extra information, if necessary. The configuration phase organizes and configures all shared and dedicated network resources. Then, it activates the slice. In the run phase, PRIMUS manages the network traffic transmission and observes the communication services and resources. If any application requirements are compromised, changes may be made, such as the virtual route used, slice topology or capacity, associating or disassociating virtual network functions. These changes are intended to improve slice performance and do not harm the application and the end-user. Finally, the deactivation phase makes available used network resources. Then, the created slice no longer exists [5].

#### 4.2.3. Resource Management Module

It manages the virtual resources of the network infrastructure and determines the association of resources to the slices. This association is based on the request received from the other PRIMUS module. To be aware of available and utilized virtual resources, resource management communicates with the NFV, which controls the creation of virtual network functions. NFV controls the network’s physical and virtual resources by analyzing and controlling VNFs, as illustrated in Figure 3. Hence, the PRIMUS allocates the necessary network resources for each application. This resource allocation comprises VNFs that establish better end-to-end routes. Applications with strict requirements, such as intensive care monitoring, are given priority over network resources. When there are insufficient or available network resources, the resource management analyzes the slice types running to delete, share resources, or split traffic across different routes to free up new resources for the restricted application.

The resource management computes performance metrics, such as packet loss rate and latency, to get information about NFV performance. Finally, PRIMUS has two main types of control messages: announce and monitor, as shown in Table 3. Both control messages assist in controlling and establishing the slice. Announce messages inform the application type and its requirements, based on identifying our previous work [21]. This message is essential to announce s-health applications that require URLLC slice type. In contrast, the monitor message updates the information about network resources availability. Thus, PRIMUS changes information about sharing, availability, and allocation of network resources through monitor control messages. The PRIMUS modules receive updates by such messages. Both control messages follow the TCP format, and they are exchanged between PRIMUS modules on a predetermined network port. Therefore, PRIMUS controls the network resources allocation and synchronizes the information.

## 5. Performance Evaluation

**Figure 5:**
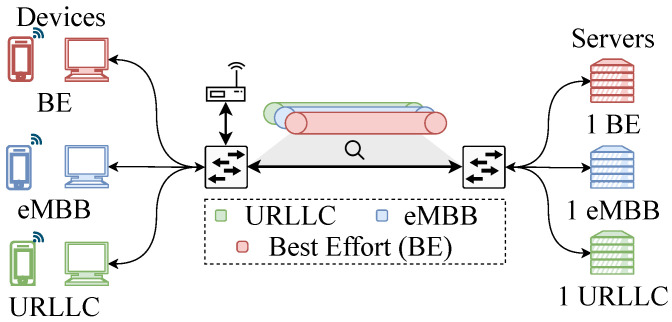
Emulation scenario.

The PRIMUS architecture was evaluated on Mininet-WiFi Network Emulator [22] that supports virtualization techniques (SDN and NFV) and network slicing. The emulator runs on the operational system Linux Ubuntu 18.04 LTS over a virtual environment (VirtualBox tool) with Intel Core i7 8500H processor (Santa Clara, California) and 8 GB of RAM. The emulation comprises of an emulated wireless network infrastructure containing two switches, one access point, three wired devices, three wireless devices, and three servers, as shown in Figure 5. The network was virtualized and sliced based on Openflow rules by Libera Hypervisor [23], and the tenants’ slices were managed by ONOS based SDN controllers [50]. The six devices are divided among the three network service/slice types (SST): URLLC, eMBB, and Best Effort (BE). Each device generates synthetic traffic simulating the specific SST to a server by iPerf tool v2.0.5 (iPerf Tool: https://iperf.fr/. Accessed on July 2020). One way latency was captured by Python scripts using the Scapy v3.7 library. We call each SST as its corresponding traffic, i.e., URLLC, eMBB, and Best Effort slices for each bit of traffic.

The evaluation scenario allows the management of slices and bandwidth use. Hence, we have compared the network performance over two scenarios: (i) considering network slicing isolation and SST priority (called **NSI scenario**), and (ii) without slicing isolation (called **no-NSI scenario**). The NSI scenario implements the slicing isolation with one specific slice for each slice type (SST), following a priority queue where URLLC has the highest priority. In contrast, the no-NSI did not implement isolation. All traffic generated compete to access the same slice (one link). The network slices compete with each other because they share the same communication physical links. Hence, 1 Mbits/s, 15 Mbits/s, and 15 Mbits for URLLC, eMBB, and BE, were generated, respectively. For BE, traffic was created threads of traffic generation to increase the competition and the amount of traffic.

In the scenarios, by the iPerf tool, all devices start the workload generation together over 100 s. As PRIMUS mechanism aims to achieve s-health requirements, we have considered network traffic similar to s-health applications. The characteristics of eMBB slice traffic correspond to telemedicine application, whereas the characteristics of the URRLC slice traffic corresponds to critical care monitoring, as shown in Table 2. At every 0.3 s interval, network traffic was generated. Moreover, we have generated best effort traffic to massive concurrent traffic to consider a more realistic scenario. Hence, the URLLC traffic has a lower throughput compared to the eMBB and BE throughput [6]. However, the URLLC requires stringent requirements than other slices. Thus, the tenant responsible for the URLLC service requested to network hypervisor for higher priorities in the processing of OpenFlow rules. We have computed the average TCP latency packet loss rates as the performance metric. The one way latency was computed by the difference between the timestamp of the message received by the server and sent by the host (received_timestamp−sent_timestamp). We have extracted the metrics from the three servers that receive the traffic generated by the devices. These metrics were measured using scripts in Python v2.7 with Scapy library. Each plotted result is an average of 33 repetitions for both scenarios. Results consider the latency metric.

### Results

The results comprise both scenario settings, NSI and no-NSI scenarios, with and without network slicing isolation. Moreover, in both scenarios, different values for bandwidth were considered. The bandwidth evaluated was 100 Mbit/s and 1 Gbit/s aiming to evaluate network slicing and PRIMUS in low and high competitive resource scenarios. The results employed network traffic from six devices competing for the same slice (no-NSI) and separated on three network slices (NSI) in the emulated network environment. Network traffic from the same slice type competes to transmit the traffic in the slice. These scenarios assist in showing the network slicing efficiency on isolating and controlling the physical network to share the resources, even in stringent concurrent scenarios. Hence, results are presented and discussed following a critical performance analysis.

**Figure 6:**
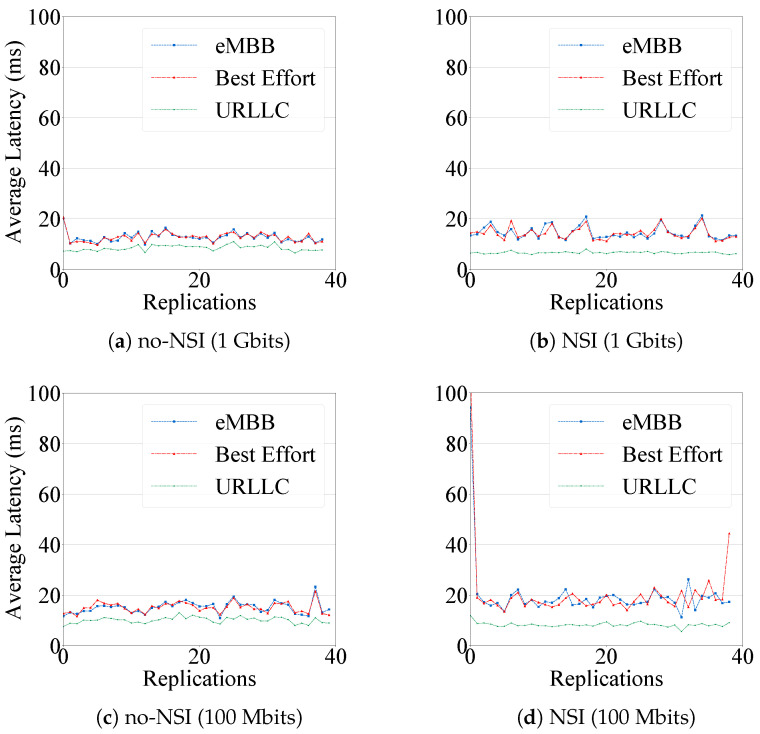
Average Latency.

Figure 6 presents the results for latency on both scenarios. Particularly, Figure 6a,b show the average latency with 1 Gbit/s of bandwidth for scenarios with and without network slicing isolation. For all slice types, the latency has reached values around 10 ms and 25 ms since there is enough bandwidth to transmit all network traffic. In the NSI scenario, the URLLC traffic has reached better latency results, such as 8 ms, than in the no-NSI scenario once the first one implements network slicing isolation and priority. eMBB and BE have reached values higher in NSI than in no-NSI. However, they have still presented low-latency values, e.g., 15 ms and 18 ms. Figure 6c,d present the average latency with 100 Mbits/s of bandwidth, i.e., more congested scenarios. The average latency for both scenarios, NSI and no-NSI, achieved similar results as the 1 Gbits/s analyses. However, PRIMUS has achieved lower latency values for URLLC in NSI scenario, such as 5 ms.

**Figure 7:**
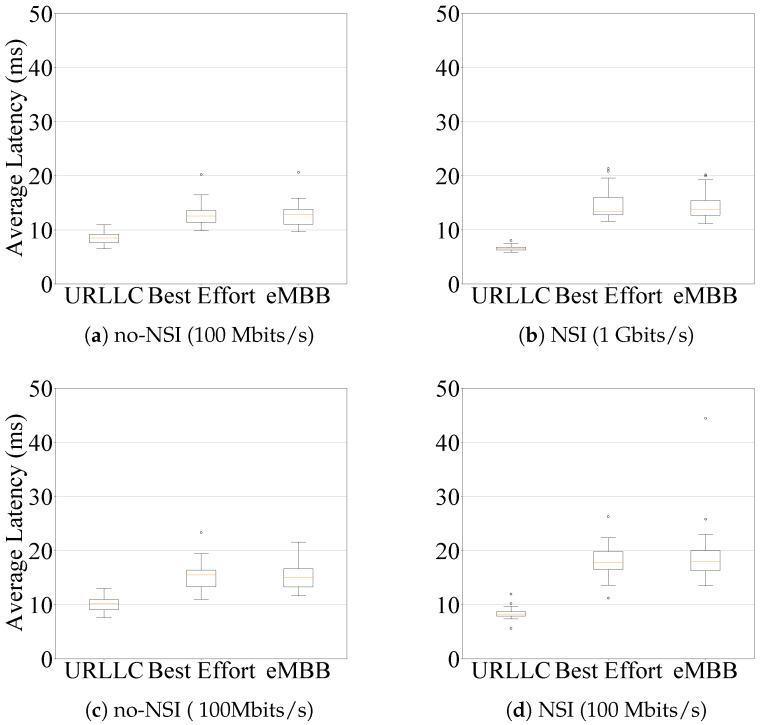
Average latency.

More details on the improvements in latency results are shown in Figure 7, considering the same scenarios and bandwidth values. Particularly, Figure 7a,b show the average latency on NSI and no-NSI with bandwidth available, and Figure 7c,d present the average latency in congested scenarios. While BE and eMBB traffic achieved latency values around 15 ms and 20 ms, URLLC reached 6 ms. In a stringent scenario without available bandwidth, the benefits for URLLC are clear. In Figure 7d, the URLCC traffic presented average latency values lower than 9 ms, while eMBB and BE reached values higher than 20 ms. Therefore, PRIMUS improved the average latency of URLLC, not harming other network traffic considerably. Moreover, URLLC did not present network packet losses.

Based on the results, PRIMUS was able to manage network slicing, network resources availability, and application requirements. Results have met low-latency even with different applications transmitting data simultaneously and stringent concurrent traffic. The high data rates transmitted on the physical link with restricted bandwidth and physical bottleneck did not interfere with PRIMUS performance. Although PRIMUS has achieved low-latency results, improvements are still necessary because the URLLC slice type demands only 1 ms of latency. However, such latency depends on efficient network physical infrastructure. Even in our network scenario, PRIMUS reached less than 125 ms of latency (value required by daily s-health applications). It is necessary to research latency since there is a delay for the controller to interact with other virtualized components. Moreover, differently from the literature, PRIMUS contributes to the implementation of a virtualized scenario with network slicing. The virtualized implementation is a potential solution to apply in the 5G/6G environment since it uses network slicing. For future analysis, a realistic smart hospital scenario containing many devices can be considered to evaluate PRIMUS scalability. However, the network slices and network traffic management used in PRIMUS will assist in network traffic concurrence since, in our results, the slice isolation improved traffic management. Therefore, the results represent success in interrelating the set of network components by automatically analyzing and managing the network resources. This work contributes to the state-of-the-art offering a virtualized scenario implementation with network slicing, network traffic management, and improvements on performance requirements to support s-health and 5G networks. However, there are open challenges, such as reducing latency, improving reliability, and validating the scalability considering different combinations of various s-health applications and more devices.

## 6. Conclusions

This article presented PRIMUS, an architecture for performance management based on adaptable smart healthcare application requirements and network resource slicing. Different from the literature, PRIMUS adapts network virtual resources and routes according to the s-health demands. Hence, it identifies the applications and configures the network on-the-fly. The results obtained in a virtualized environment, supporting SDN, NFV, and network slicing, show the feasibility to achieve the performance requirements of s-health. PRIMUS achieved latency values less than 10 ms in URLLC slice type (refers to critical care monitoring), and less than 20 ms for eMBB (refers to telemedicine). The required 1 ms of URLLC latency can only be achieved with network physical infrastructure efficient, such as 5G and 6G networks. Hence, this work contributes to the state-of-the-art and research community by implementing a virtualized scenario considering network slicing, network performance management, and critical applications performance requirements, i.e., s-health applications. As future directions, we intend to improve reliability, latency values considering a 5G environment, and PRIMUS scalability with more devices. Therefore, we concluded that network slicing is absolutely necessary to enable the smart healthcare envisioned scenario in 5G and 6G networks. Furthermore, network slicing with virtualization techniques will be key technologies to resolve heterogeneity issues.

## Figures and Tables

**Table 1:** Main works of Network Performance Management.

Reference	Approach					Requirement			Implementation				
	Reso.Alloc.	Traf.Shaping	Traf.Separ.	Scheduling	Netw.Slicing	Reliability	Latency	Others	Simulation	Experiment	Mathemat.Mod.	Abstract Level	Virtualization
Clark et al. [25]		✓						✓		✓	✓		
Lloret et al. [14]			✓			✓	✓		✓				
Fu et al. [26]			✓					✓				✓	
Uddin et al. [27]			✓					✓	✓			✓	✓
Vergutz et al. [13]			✓	✓			✓		✓				
Li et al. [28]				✓			✓		✓				
Petrov et al. [15], Wang et al. [16]				✓		✓				✓	✓		
Ge et al. [17], Kalor et al. [29]				✓	✓	✓	✓				✓		
Yilmaz et al. [30], Karimi et al. [31]				✓	✓	✓	✓		✓				
Kurtz et al. [18], Bouzidi et al. [32]	✓			✓	✓		✓		✓				✓
Sciancalepore et al. [33,34]		✓		✓	✓			✓	✓				
Salhab et al. [35]	✓			✓	✓			✓		✓			
Richart et al. [36]	✓			✓	✓			✓	✓				
Salvat and Marquez et al. [37,38]	✓				✓			✓	✓	✓	✓		
Husain et al. [19]					✓	✓	✓					✓	

**Table 2:** S-Health and performance requirements.

Application Type	Latency	Bandwidth	Reliability
Telemedicine	≤10 ms	≥200 Mbps	-
Remote Surgery	≤1 ms	10 Mbps	99.999%
Diabetes/Temperature	≤125 ms	10 Mbps	-
Emergency Ambulance	≤1 ms	10 Mbps	99.999%
Critical Care Monitor	≤1 ms	10 Mbps	99.999%
Remote Monitor	≤10 ms	≥50 Mbps	-
Smart Medication	≤125 ms	≥20 Mbps	-

**Table 3:** PRIMUS control messages.

Control Message	Content	Description
Announce	Application type	Inform if application
	and requirements	is s-health
Monitor	Network resources	Inform about network
	(e.g., resources use)	resources availability
